# Pigment Penetration Characterization of Colored Boundaries in Powder-Based Color 3D Printing

**DOI:** 10.3390/ma15093245

**Published:** 2022-04-30

**Authors:** Danyang Yao, Jiangping Yuan, Jieni Tian, Liru Wang, Guangxue Chen

**Affiliations:** 1 State Key Laboratory of Pulp and Paper Engineering, South China University of Technology, Guangzhou 510640, China; dyyao9722@163.com (D.Y.); tianjieni123@126.com (J.T.); wanglir0510@163.com (L.W.); 2 College of Communication and Art Design, University of Shanghai for Science and Technology, Shanghai 200093, China; yuanjp@usst.edu.cn

**Keywords:** color 3D printing, penetration depth, chromaticity, image-based metric, color reproduction

## Abstract

Color 3D printing has widely affected our daily lives; therefore, its precise control is essential for aesthetics and performance. In this study, four unique test plates were printed using powder-based full-color 3D printing as an example; moreover, the corresponding pigment-penetration depth, chromaticity value and image-based metrics were measured to investigate the lateral pigment penetration characteristics and relative surface-color reproduction of each color patch, and to perform an objective analysis with specific microscopic images. The results show that the lateral pigment-penetration depth correlates with the number of printed layers on the designed 3D test plates, and the qualitative analysis of microscopic images can explain the change in chromaticity well. Meanwhile, there is an obvious linear correlation between the mean structural similarity, color-image difference and color difference for current color samples. Thus, our proposed approach has a good practicality for powder-based color 3D printing, and can provide new insight into predicting the color-presentation efficiency of color 3D-printed substrates by the abovementioned objective metrics.

## 1. Introduction

Three-dimensional printing is a layered manufacturing process based on the principles of additive manufacturing and rapid prototyping [1]. It is based on digital model files and uses bondable materials such as plastic, gypsum powder, paper sheet, and resin to manufacture three-dimensional objects by printing layer by layer and overlaying different shapes in a continuous layer-forming method [2], which also contains color replication [3]. With the continuous development and upgrading of 3D printing materials and equipment with a rapid growth in industry scale, color 3D printing technology has become a key branch of the 3D printing industry [4].

Based on the printing materials, color 3D printing can be divided into six major categories, namely, plastic-, paper-, powder-, bio-, food- and metal-based color 3D printing [5]. There are two ways for color 3D printing to achieve a lifelike surface color: one is to add a coloring stage to the post-printing process [6,7]; the second is to introduce the color discretization of graphic printing into 3D printing during the forming process. The latter method directly provides richer color reproduction and is called full-color 3D printing with accurate digital colorization arrangement [8,9].

Color is one of the most expressive and attractive surface attributes in the 3D world, and high-precision color reproduction is something that all categories of 3D printing technology aspires to achieve at this stage. It is even more essential in the custom production and cultural artwork industries [10,11]. Powder-based 3D printing, also known as inkjet 3D printing technology (3DP), was one of the first technologies proposed to enable full-color 3D printing [12]. However, at this stage, 3D-printed products still cannot fully meet the individual needs of users [13], and color reproduction research still lacks a unified theoretical and standard numerical model for predicting the color quality of 3D prints [14].

Currently, color quality evaluation of full-color 3D printing is a focus of interest for color researchers and a key part of the industrialization process of full-color 3D printing. Lu et al. proposed the graded solution method for ink-permeation pressure distribution and established a spatio-temporal evolution equation for ink-permeation pressure distribution based on experimental parameters. This study analyzed the ink permeation behavior only from the horizontal plane, without taking into account the sides (cross-sections) of the samples [15]. Yang et al. proposed a fast slicing and color-acquisition algorithm for 3D models to obtain color information coordinate formulas and calculate axis pixel-information coordinates for color 3D printing, which achieved the expected purpose with a high efficiency. However, the degree of refinement was somewhat lacking [16]. Li et al. used EPSON DX-5 for spontaneous color 3D printing; full-color printing was implemented based on halftoning algorithms that used different threshold matrices for different ink channels; the performances of the various algorithms were evaluated in terms of both subjective and objective indices. The calculation process was more complicated [17]. In the study of the penetration characteristics of the pigments on the lateral interface (cross-section) of 3D prints and their surface color features, there is an urgent need for color quality prediction for full-color 3D printing.

In addition, there has also been related progress in research on the quality evaluation of color reproduction for 3D-printed surfaces. Wang et al. proposed a microstructure image analysis method for powder-based 3D-printed samples postprocessed by different impregnating agents to determine the optimal process parameters. The effect of color on 3D samples has not been considered in detail [18]. Yuan et al. studied the color reproduction of specific 3D samples produced by plastic-based color 3D printing, using a linear correlation analysis between color difference and image-based metrics, including a mean structural similarity (MSSIM) and color structural similarity (SSIM) map. There was no consideration of the effect of pigment penetration behavior on color reproduction [19]. Tian et al. skillfully explored the quantitative correlation between the color feature of a paper-based 3D-printed coloring layer and its blank layer attached underneath using ∆E*_ab_, the feature similarity index measure of color images (FSIMc), and an improved color image difference (iCID). This study ignored the problem of 3D sample boundary coloring [20]. Therefore, image-based metrics have mature applications in the evaluation of the color-reproduction quality of color in 3D printing as efficient numerical visualization tools.

In fact, the color management in the classic graphic printing domain performs badly in adjusting the color reproduction of color 3D printing because the pigment penetration behavior changes significantly [4]. Thus, combined with image-based metrics and color differences, this paper uses a powder-based color 3D printer and a specific experimental model to analyze the characteristics of internal pigment penetration, as well as quantitative quantification to enrich the color-reproduction control of color 3D printing. By pre-designing the test structure, the color 3D-printed sample was cut with certain thin pieces, and the microscopic imaging method was used to quantify the depth of the pigment penetration in the sides of the pieces. The current characterization of pigment penetration characteristics can provide more accurate factual bases and analysis methods for accurate color-reproduction prediction in color 3D printing.

The structure of this study is divided into four parts: The first part contains the background of this study, previous research results and problems, and the purpose and significance of this research study. The second part contains the model design as well as the experimental method used. The third part analyzes the experimental results from three perspectives. The fourth part contains the results and discussion.

## 2. Materials and Methods

**Figure 1:**
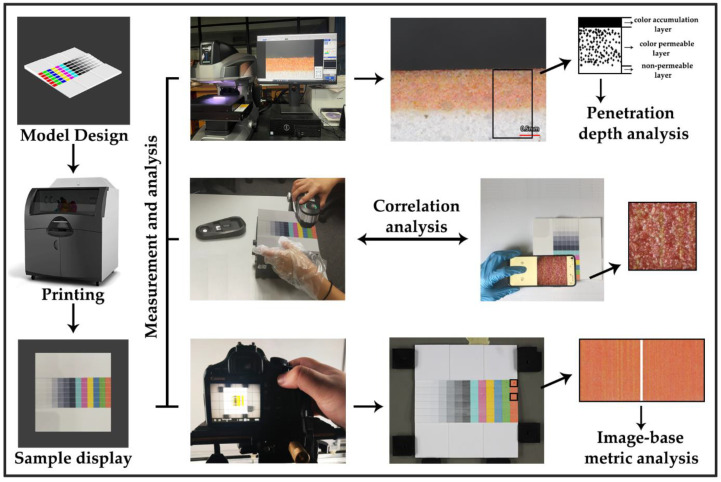
The experimental framework with relative equipment.

The experimental framework of this paper is shown in Figure 1. Firstly, four specific 3D test models were designed using the 3D software Autodesk 3ds Max (San Rafael, CA, USA), and samples were printed using the 3D System Z860 printer (Rock Hills, SC, USA). Next, a 3D profilometer VR-5000 (Osaka, Japan) was used to obtain the surface pigment penetration depth; the spectrodensitometer X-Rite i1Pro2 (Grand Rapids, Michigan, USA) was used to measure the chromaticity values (L*a*b*) of the surface color; a TIPSCOPE Mobile Microscope (Hubei, China) was used to obtain the microscopic surface; and a Canon EOS 500D camera (Shimomaruko, Ota-ku, Tokyo, Japan) was used to acquire high-resolution images of surface color patches to calculate image-based metrics.

### 2.1. Model Design

Four 3D test models were designed. The center area of this model had six thicknesses of coloring stairs, and its two sides had three thicknesses of white blocks as the blank control. Each test model included thirteen color samples and six coloring stairs. The color sample was selected from six primary colors, r (red), g (green), b (blue), c (cyan), m (magenta), y (yellow), and neutral colors k (black), 0.8k, 0.6k, 0.4k, 0.2k, 0.1k, and w (white) with specific order changes, where 0.1k means 10% black, expressed as a decimal for subsequent marking, and so on. In Table 1, for each color sample, specific CIEL*a*b* values are given independent of the device presentation. In Table 2, specific parameters for different stairs are given for each model, where these four test samples are named I, II, III, and IV in turn. C_s1_ denotes the thickness of the first step of the color layer, W_s1_ denotes the thickness of the first step of the white layer, and so on for the others.

### 2.2. Pigment Penetration Depth, Chromaticity Measurement and Image Acquisition Quantification

Using a 3D profilometer (its magnification = 80×) to obtain the depth of pigment penetration on the lateral interface of each color patch on the 3D test plate, the thickness values were measured three times and averaged for the next analysis. The chromaticity of the color patches on each test plate was measured by a spectrophotometer (parameters: measurement condition = M1, illumination source = D50, observer angle = 2 degrees), averaged three times for each patch, and correlated with the microscopic images acquired using TIPSCOPE. In the standard imaging system, the camera focus was set at the same horizontal height as the center point of the color 3D test plate to obtain the microscopic imaging for each color patch. Then, further objective quantitative evaluations were performed in a compiled MATLAB program using the feature-similarity index measures of color image (FSIMc), mean structural similarity (MSSIM) and color image difference (iCID) metric algorithms.

### 2.3. Data Analysis

In this study, the degree of pigment penetration on the side of the color patches, as well as the chromaticity and similarity between the blocks, were calculated and analyzed.

(1) $$\mathrm{Height}=H_{i}-h$$

where h is the color block surface color thickness preset value (h = 200 μm), H_i_ is the penetration depth value of the i color patch surface color, and i is a positive integer from 1 to 6.

(2) $$\Delta E_{76}^{*}=\sqrt{{\left(L_{i}^{*}-L_{1}^{*}\right)}^{2}+{\left(a_{i}^{*}-a_{1}^{*}\right)}^{2}+{\left(b_{i}^{*}-b_{1}^{*}\right)}^{2}}$$

where ΔE*_76_ is the relative chromaticity; L*_1_, a*_1_, and b*_1_ are the color of the first stair color patch; L*_i_, a*_i_, and b*_i_ are the color of the i stair color patch; and i is a positive integer from 2 to 4.

(3) $$\mathrm{FSIMc}=\frac{\sum_{\Omega }S_{\mathrm{PC}}\left(x\right)\cdot S_{G}\left(x\right)\cdot {\left[S_{I}\left(x\right)\cdot S_{Q}\left(x\right)\right]}^{\lambda }{\mathrm{PC}}_{m}\left(x\right)}{\sum_{\Omega }{\mathrm{PC}}_{m}\left(x\right)}$$

For these, see Ref. [21].

(4) $$\mathrm{MSSIM}\left(x,y\right)=\frac{\left(2\mu_{x}\mu_{y}+C_{1}\right)\left(2\sigma_{\mathrm{xy}}+C_{2}\right)}{\left(\mu_{x}^{2}+\mu_{y}^{2}+C_{2}\right)\left(\sigma_{x}^{2}+\sigma_{y}^{2}+C_{2}\right)}$$

where μ_x_, μ_y_, σ_x_, σ_y_, and σ_xy_ are the x-image mean, y-image mean, x-image variance, y-image variance, and two-image covariance, respectively, and C_1_ and C_2_ are nonzero small constants avoiding zero in the denominator.

(5) $${\mathrm{iCID}}_{A}\left(S,T\right)=1-\frac{1}{\left|A\right|}\sum_{i\in A}\left[l_{L}\left(s_{i},t_{i}\right)\cdot c_{L}\left(s_{i},t_{i}\right)\cdot s_{L}\left(s_{i},t_{i}\right)\cdot l_{C}\left(s_{i},t_{i}\right)\cdot l_{H}\left(s_{i},t_{i}\right)\cdot c_{C}\left(s_{i},t_{i}\right)\cdot c_{C}\left(s_{i},t_{i}\right)\right]$$

For these, see Ref. [22].

## 3. Results and Analysis

### 3.1. Effect of the Number of Layers Printed on the Surface of Powder-Based Color 3D Test Plates on the Depth of Pigment Penetration

**Figure 2:**
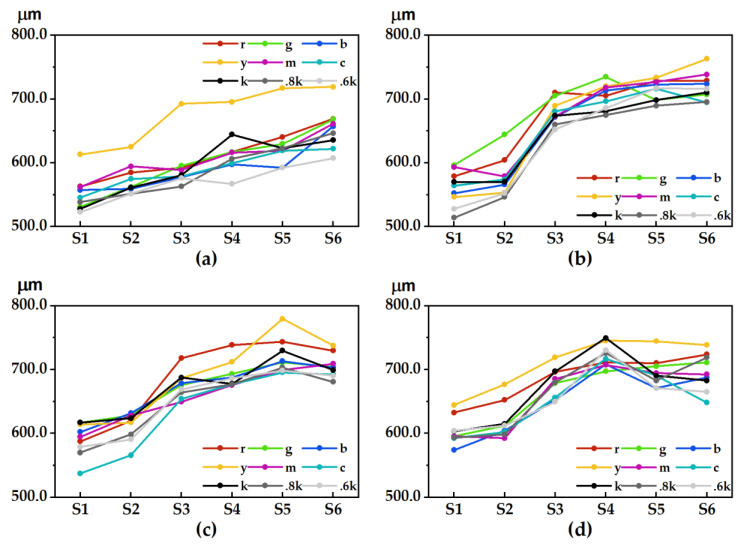
The lateral pigment penetration depth: (**a**) test plate I; (**b**) test plate II; (**c**) test plate III; (**d**) test plate IV.

In Figure 2, the penetration characteristics of the pigments on the sides of four 3D test plates with different numbers of printed layers are shown. The lateral thickness value of the color sample on each stair of the 3D test plate was first compared with the color preset value at the designing stage of the test models, and the difference was calculated and noted as the penetration depth. Considering that the bottom layer of the test plate is noncolored, the measurement targets select primary color samples and gray color samples, including k, 0.8k, and 0.6k. For the sake of image layout, “0.8k” is abbreviated to “.8k”, and so on.

Except for test plate I, the general trend of the penetration depth of the lateral pigments of the other test plates showed a consistent change, and the higher the number of printed layers was, the greater the penetration depth of the lateral pigments of the test plate was. However, when the number of printed layers reached a certain height, the change in their penetration depth tended to level off or slightly decrease. Test plates II and III on stair 1 to stair 3 show that the sample penetration degree grows more quickly and then relatively slowly. However, the penetration depth at stair 6 was relatively obvious for each color sample, such as color sample g and color sample c in test plate II, most of the color samples in test plate III, and color sample c in test plate IV, which all had obvious decreasing trends. Compared with the neutral gray sample, the depth of penetration of the color-sample side was more obvious. For example, the penetration depths of color samples r, g, y and m in test plate I were significantly greater than those of color sample 0.6k, the penetration depths of color samples r and g in test plate II were greater than those of color sample 0.8k, the penetration depths of color samples r and y in test plate III were greater than those of color sample 0.8k, and the penetration depths of color samples r and y in test plate IV were greater than those of color sample 0.6k.

**Figure 3:**
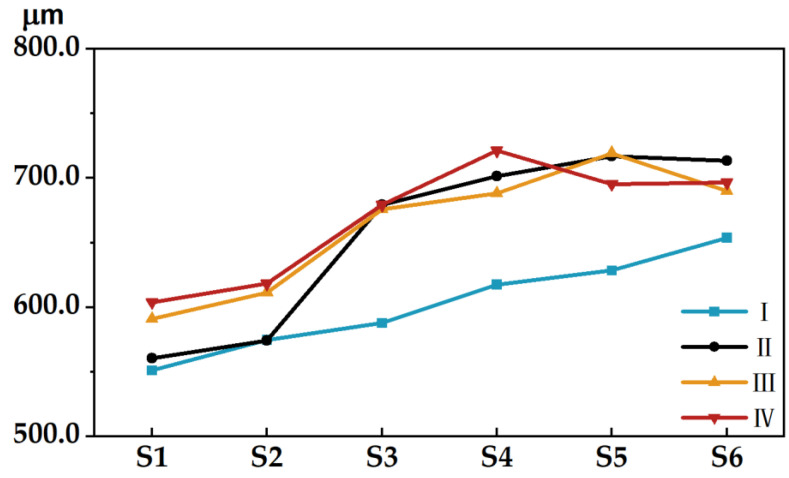
Average depth of pigment penetration on the side of each 3D test plate.

In Figure 3, by calculating the average of the penetration depths of each stair color sample on a test plate, the influence of different printing layers on the surface of the test plate on the degree of pigment penetration became more obvious, and the higher the number of printing layers on the test plate was, the more obvious the degree of penetration was. For example, the average penetration depth of each stair color sample in test plates III and IV is significantly higher than that of plate I. When the number of printed layers is low, the average depth of penetration of each color sample on the surface of the test plate grows faster, but after reaching a certain height, the average depth of penetration is basically similar; however, there is a significant decline in the highest stair. The average depth of penetration in test plate III increases abruptly after stair 3. It is hypothesized that the thickness of the plate base at stair 3 reaches a certain height value and that the average depth of penetration shows a sudden change, which has a strong relationship with the thickness of the plate base.

### 3.2. Effect of the Number of Layers Printed on the Surface of the Powder-Based Color 3D Test Plate on Color Chromatic Aberration

**Figure 4:**
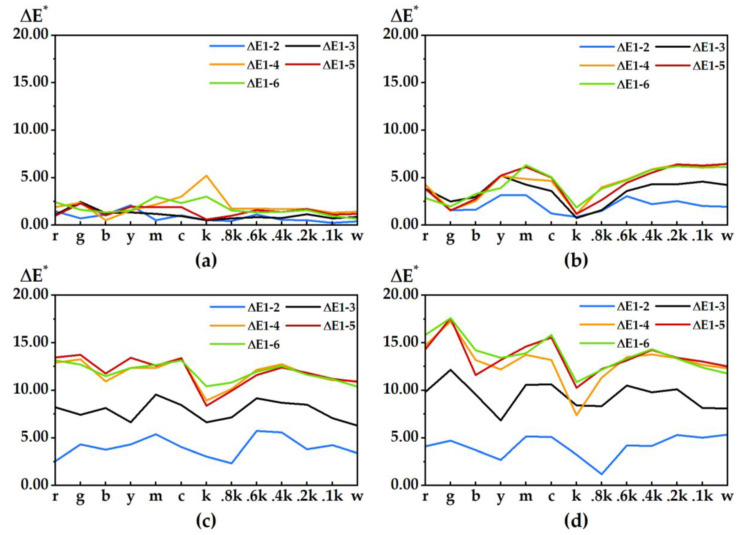
Three-dimensional test plate surface chromaticity: (**a**) test plate I; (**b**) test plate II; (**c**) test plate III; (**d**) test plate IV.

In Figure 4, the chromaticity between the color sample on coloring stair 1 of the 3D test plate and the color samples at the same position on the other stairs is shown. Overall, it can be seen clearly that the chromaticity varies significantly depending on the number of layers printed on the test plate and is positively correlated with the number of layers printed. Except for the 3D test plate I, the trend of chromaticity between each coloring stair and stair 1 in the rest of the plates is basically the same—that is, the stair contains the increase in the number of printed layers, where the chromaticity values at stair 2 and stair 3 fluctuate greatly. For example, the chromaticity between stair 4 and stair 6 in test plate I and stair 1 fluctuated more, while the rest of the stairs changed very little and tended to be the same, whereas the other three test plates and test plate I generally showed the opposite trend. The chromaticity of the color sample fluctuated more than the chromaticity trend of the noncolor sample. For example, the fluctuations of color samples g, m, c, and k in test plate I; color samples y, m, and c in test plate II; color samples r, g, y, m, and c in test plate III; and color samples g, m, and c in test plate IV were all relatively obvious. Compared with the other color samples, color sample k showed a large fluctuation in chromaticity when the number of printed layers was small, but when the number of printed layers reached a certain height, the change was relatively small.

**Figure 5:**
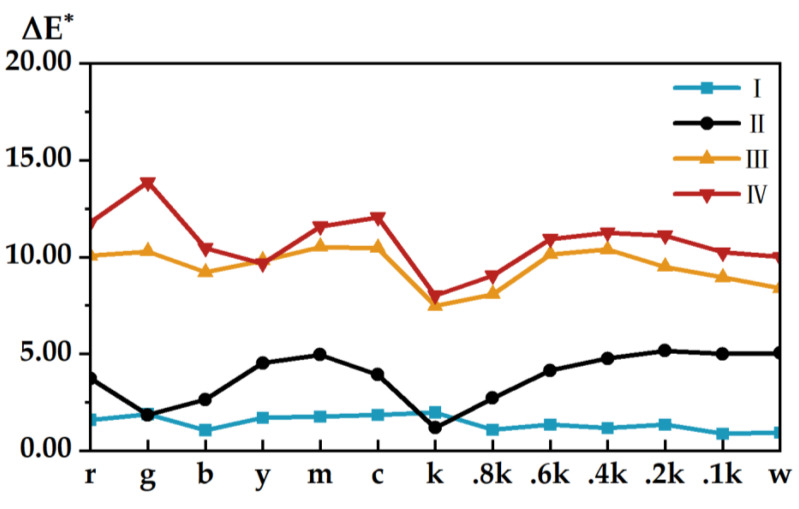
Three-dimensional test plate surface color average chromaticity.

**Figure 6:**
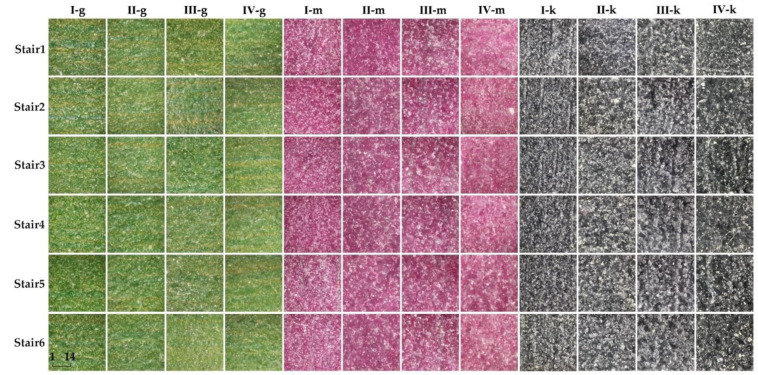
Comparison of microscopic images of color samples g, m and c on different test plates.

Figure 5 shows the average chromaticity of each color sample on the 3D test plate, and it can be observed that the fluctuation of each color sample varies widely. Except for test plate I, the overall trend of the other test plates is consistent. When the number of printed layers reaches a certain value, the average chromaticity values among the color samples on the test plate change more similarly. For example, the average chromaticity changes of color samples y and k in test plate III and test plate IV are basically the same. In addition, the average chromaticity values of g, m and c in color samples and 0.6k and 0.4k in noncolor samples are relatively large compared to other color samples. Figure 6 analyzes the chromaticity from the perspective of microscopic imaging on the surface of the color sample to explain the fluctuating characteristics of the chromaticity-variation trend, in which the vertical arrangement is the microscopic images of the color patches from coloring stair 1 to stair 6 on the test plate, in order, and the horizontal arrangement is the color samples g, m and k on the four test plates, in order. I-g indicates the color sample g on test plate I, and so on for the others. All diagrams are used is a small paste microscope, and the scale is 1:14 (when using it needs to be pasted on the phone lens, with the magnification of up to 400×).

In Figure 6, due to the problem of a lack of space, only the color samples g, m and k with large fluctuations in chromaticity in Figure 4 were selected. The brightness of the colors of the 3D test plates with different printing layers were largely varied, and the color samples in test plates II, III and IV were obviously darker than those in plate I. The color of different coloring stairs in the same test plate also has a certain difference; the higher the coloring stair is, the less bright the color sample is compared to the lower stair of the color sample. Test plate I in the colored sample k contains white particles compared to the colored samples g and m, so its chromaticity value fluctuation is relatively large. The color of sample k on different stairs in test plate II is similar. Except for test plate I, the color samples g, m and k on stair 5 and stair 6 on the other test plates have very little difference in color, while the rest of the color samples on the stairs have a relatively low brightness.

### 3.3. Image-Based Metrics Analysis of Surface Coloring Stairs on Powder-Based Color 3D Test Plates

**Figure 7:**
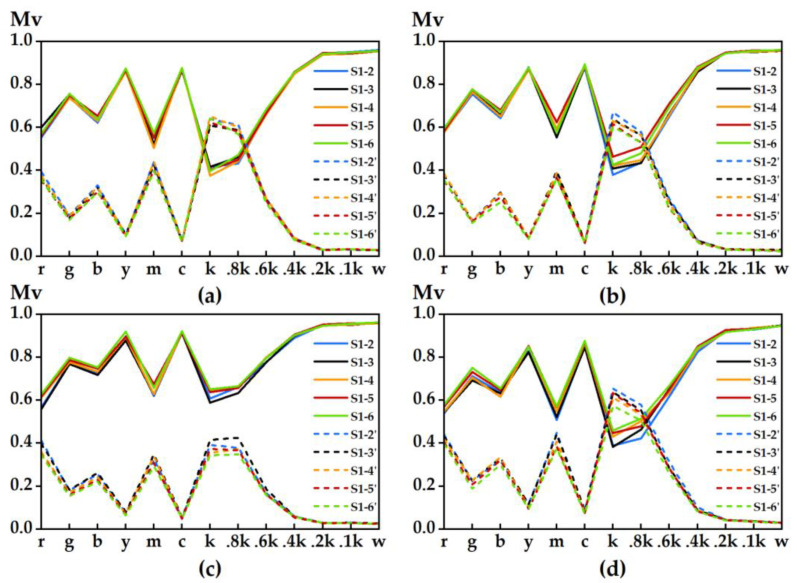
Similarity between color samples: (**a**) test plate I; (**b**) test plate II; (**c**) test plate III; (**d**) test plate IV. Solid lines S1-2 indicate MSSIM image-based metric curves, while dashed lines S1-2′ indicate iCID image-based metric curves.

Figure 7 shows the similarity between the high-definition color samples on the 3D test plate of coloring stair 1 and the color samples at the same position of the other stairs. The top and bottom parts of the figure are selected based on the MSSIM metric and the iCID metric obtained from the MATLAB compilation, respectively, where the more similar the color samples, are the closer the MSSIM curve value is to 1, and the closer the iCID curve value is to 0, the smaller the difference between color samples is. The vertical coordinate Mv (metric value) represents the change in the value of the image basis metric. In general, the degree of similarity and the degree of difference between the color samples of the 3D test plates with different numbers of printed layers are basically the same, and the similarity and difference curves of the color samples on different coloring stairs are also very consistent for the same test plate. Overall, the similarity between coloring stair 6 and stair 1 is slightly better than that of other coloring ladders, e.g., color samples g, y and c in the four test plates. Color sample areas fluctuate more and the differences between them are more obvious, for example, in color samples r, b and m. Color samples 0.2k, 0.1k and w in noncolor areas fluctuate steadily. However, for the color samples in the transition region, the degree of difference is larger, for example, in color samples k, 0.8k and 0.6k, probably due to the large differences in color contrast between white particles and noncolored adhesives in the color samples at the transition region.

**Figure 8:**
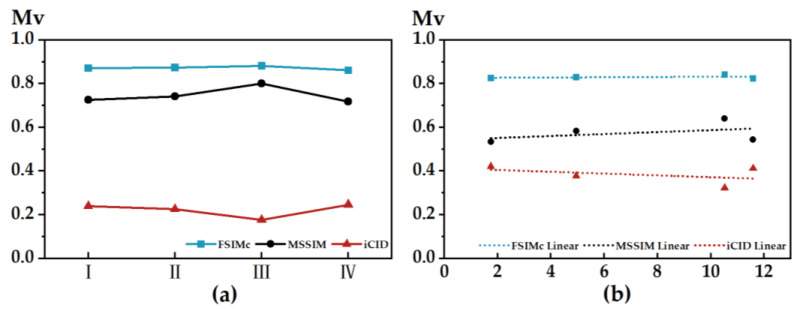
(**a**) Quantitative correlation of image basis metrics; (**b**) image basis metrics and chromaticity linearization.

The degree of similarity between color patches can be observed in several ways. The left panel in Figure 8 shows the quantified correlation between the three image-basis metrics compiled based on MATLAB, in which the trends of the image-basis metric based on FSIMc and MSSIM are roughly the same, and the FSIMc curve is more stable overall; the MSSIM value rises and then falls, the iCID value falls and then rises, and the sum of the two values approximately equates to 1 but is not equal to 1. The right panel shows the quantified relationship between the chromaticity of color sample m and the image basis metrics (only a single color sample is selected for space). The chromaticity of the color sample on the test plate shows a stable positive correlation with the objective index FSIMc and MSSIM values and a negative correlation with the objective index iCID value.

## 4. Discussion and Conclusions

In this study, we studied the pigment penetration characteristics on the lateral interface (cross-section) of the test plate and then combined the correlation between the chromaticity measurement and the surface microscopic images, as well as the objective assessment of acquired image quality, to provide the color reproduction evaluation based on powder-based color 3D printing. The powder-based color 3D print is made of white powder particles bonded with an adhesive. When its surface powder particle arrangement is not dense, this affects the vertical penetration of the surface-color adhesive, thus indirectly affecting the sample surface-color reproduction [23,24].

The lateral pigment-permeation distribution of the powder-based color 3D printing test plate directly affects its boundary-coloring accuracy and indirectly affects the surface-color rendering. The pigment penetration on the side of the substrate helps to analyze the intrinsic causes of the trends at the microscopic level. For a test plate with different printing layers, the penetration depth of lateral pigment has a good constant similarity and generally tends to rise with the number of printing layers [25], but significantly declines after reaching a certain height. From this, the test plate boundary coloring can be predicted.

Although chromaticity measurement can quickly predict the color reproduction quality of a flat color patch, the surface properties of the 3D-printed underlying media always change with non-negligible fluctuations. Therefore, the color reproduction of the underlying media surface is monitored and correlated with the microscopic image of the printed surface, and it is obvious from the correlation between chromaticity and the microscopic image that the chromaticity of the color samples on the surface of the underlying media fluctuates more, while the noncolor color samples are more flat. The chromaticity fluctuation also becomes larger with the increase in the number of printed layers [26,27]. The average chromaticity is correlated with the image-based metrics to improve the color reproduction prediction under different objective metrics. Thus, based on the modeling idea of the paper-coloring efficiency formula in the field of graphic printing, the formula of the surface-coloring efficiency metric for color 3D-printed substrate should be further investigated by other researchers. Overall, our proposed approach is of good practicality for lateral pigment penetration analysis.

## Figures and Tables

**Table 1:** Color parameters in each sample patch.

Color	r	g	b	y	m	c	k	0.8k	0.6k	0.4k	0.2k	0.1k	w
L*	54	88	30	98	60	90	0	21	43	63	82	91	100
a*	81	−79	68	−16	94	−51	0	0	0	0	0	0	0
b*	70	81	−112	93	−60	−15	0	0	0	0	0	0	0

**Table 2:** Thickness parameters in each 3D test model.

ID	Substrate (cm)	Color Layer (cm)						White Layer (cm)					
		C_S1_	C_S2_	C_S3_	C_S4_	C_S5_	C_S6_	W_S1_	W_S2_	W_S3_	W_S4_	W_S5_	W_S6_
I	0.2	0.02	0.04	0.06	0.08	0.1	0.12	0	0.02	0.04	0.06	0.08	0.1
II	0.2	0.04	0.08	0.12	0.16	0.2	0.24	0.02	0.06	0.1	0.14	0.18	0.22
III	0.1	0.08	0.16	0.24	0.32	0.4	0.48	0.06	0.14	0.22	0.3	0.38	0.46
IV	0.1	0.1	0.2	0.3	0.4	0.5	0.6	0.08	0.18	0.28	0.38	0.48	0.58

## Data Availability

Data is contained within the article.
